# Burden and disutility of sleep disturbance and early morning OFF symptoms in people with advancing Parkinson’s disease: a vignette-based approach using the EQ-5D-5L

**DOI:** 10.1186/s41687-026-01053-w

**Published:** 2026-04-14

**Authors:** Josefa Domingos, Pablo Arija, Irene A. Malaty, Rajesh Pahwa, K. Ray Chaudhuri, Marco Boeri, Maja Kuharic, Anjana Lalla, Zachary Baldwin, Connie H. Yan, Marieke Heisen, Divya Mohan, Hannah Penton

**Affiliations:** 1 Parkinson’s Europe, Orpington, UK; 2 Egas Moniz School of Health & Science, Almada, Portugal; 3 Patient Centered Outcomes, OPEN Health, Rotterdam, The Netherlands; 4 Department of Neurology, Fixel Institute for Neurological Diseases, University of Florida, Gainesville, FL USA; 5 University of Kansas Medical Center, Kansas City, KS USA; 6 Kings College Hospital and Kings College, London, Kings College Hospital London Dubai, Dubai, UAE; 7 Dementech Neuroscience Clinical Academic Centre, London, UK; 8 Patient Centered Outcomes, OPEN Health, London, UK; 9 Health Economics, Queen’s University Belfast, Belfast, UK; 10 Northwestern University Feinberg School of Medicine, Chicago, IL USA; 11 BetterHealth Outcomes LLC, Chicago, IL USA; 12 AbbVie Inc, South San Francisco, CA USA; 13 AbbVie Inc, North Chicago, IL USA; 14 Patvocates, Munich, Germany

**Keywords:** Advancing Parkinson’s disease, Health state utilities, Sleep disturbance, Early morning OFF, Patient-centered care, Neurology, Vignette-based utilities, EQ-5D

## Abstract

**Background:**

Parkinson’s disease (PD) is characterized by motor and non-motor symptoms that fluctuate as oral medication wears off, causing periods when symptoms return (“OFF” time) and periods of control (“ON” time). These fluctuations often occur at night and early morning. Sleep disturbance (SD) and early-morning OFF time (EMO) have been shown to decrease health-related quality of life (HRQoL) in people with PD (PwP). However, limited evidence exists on how these symptoms impact health state utility values (HSUVs) used in economic modeling. This study aimed to estimate the burden and disutility of SD and EMO in PwP through vignettes valued using the EQ-5D-5 L.

**Methodology:**

An online survey was completed by adults (≥ 30 years) with self-reported PD diagnosis for ≥ 5 years and ≥ 2 h/day of OFF time, on oral PD medications, and residing in the US or UK. The survey included EQ-5D-5 L and EQ-VAS assessments for 4 vignettes—No SD or EMO, SD (without EMO), EMO (without SD), and Both SD and EMO—plus questions exploring the burden of SD and EMO. EQ-5D-5 L responses were converted into US utility values to estimate HSUVs and the disutility of SD and EMO.

**Results:**

Seventy-five individuals completed the survey; 52.00% were male, and 78.66% resided in the US. The average age was 64.32 years, with a mean time since diagnosis of 9.82 years and an average OFF time of 3.91 h/day. In the previous week, 96.00% and 98.67% of respondents reported experiencing SD and EMO, respectively. The presence of either SD (HSUV: 0.796) or EMO (HSUV: 0.701) alone resulted in lower EQ-5D-5 L utilities, with the presence of both valued least (HSUV: 0.528), compared with when both SD and EMO were absent (HSUV: 0.911). EQ-VAS scores ranged from 81.01 for no symptoms to 48.41 for both.

**Conclusions:**

SD and EMO impose a significant burden on PwP and decrease HSUVs. As SD and EMO were associated with significant disutility, they should be incorporated into economic models to comprehensively assess PD treatment benefits. Raising awareness among PwP and healthcare providers and prioritizing treatments that minimize SD and EMO can reduce their burden and improve HRQoL for PwP.

**Supplementary Information:**

The online version contains supplementary material available at 10.1186/s41687-026-01053-w.

## Plain language summary

**Why is this study needed?:**

People with Parkinson’s (PD) often struggle with sleep disturbances (SD) and early morning OFF (EMO) symptoms, which can seriously affect their health-related quality of life (HRQoL). It is essential to comprehensively assess the value of PD treatments, and ignoring these symptoms in economic evaluations underestimates their true impact.

**What is the key issue this manuscript addresses?:**

Health Technology Assessment (HTA) bodies such as the National Institute for Health and Care Excellence, Haute Autorité de Santé, and Zorginstituut Nederland prefer HRQoL to be measured using the EQ-5D measure to generate health state utility values (HSUVs) for use in economic evaluations. The aim of this study was to estimate HSUVs associated with SD and EMO in people with advancing PD (aPD) through vignettes valued using the EQ-5D-5L.

**What is the main point of your study?:**

SD and EMO are highly prevalent in people with aPD (PwP) and negatively affect their HRQoL. We estimated the burden and the impact of SD and EMO on HRQoL to ensure a more comprehensive assessment of treatment value.

**What are your main results and what do they mean?:**

Estimated HSUVs demonstrated the meaningful negative impact of SD and EMO on HRQoL, with EMO having greater impact than SD, as reflected particularly in the EQ-5D domains of mobility, self-care, and usual activities. Given the high prevalence of SD and EMO in PwP and their significant impact on HRQoL, it is essential to prioritize treatments that offer continuous symptom control throughout the entire day and night.

**Supplementary Information:**

The online version contains supplementary material available at 10.1186/s41687-026-01053-w.

## Background

Parkinson’s disease (PD) is characterized by a range of motor symptoms such as tremors, rigidity, and gait problems, as well as non-motor symptoms including sleep problems, drooling, and anxiety [1]. As PD progresses to advanced stages, the therapeutic effect of oral levodopa shortens, leading to motor and non-motor fluctuations—alternating periods of “ON” (symptoms controlled) and “OFF” (symptoms re-emerge). These fluctuations result primarily from long-term oral levodopa therapy combined with disease progression and are further exacerbated by delayed gastric emptying and variable intestinal absorption [2]. Managing these fluctuations with oral medications alone becomes increasingly challenging, particularly overnight and in the early morning. The wearing off of medications during the night can lead to worsening of symptoms that not only disrupt sleep and lead to poor sleep quality but can also delay the start of an individual’s day (occurrence of early morning OFF time [3] time or morning akinesia) until the morning dose of oral PD medication takes effect [3–6].

Episodes of sleep disturbance (SD) and EMO can occur in 39.43% to 59.70% of people with PD (PwP) across all disease stages, respectively, causing significant disability and substantially affecting quality of life [7–10]. PD-related SD involves difficulty initiating or maintaining sleep, frequent early awakening, nightmares, REM sleep disorder, rigidity or pain in limbs, or restlessness of limbs [11]. The most prevalent symptoms associated with EMO are bradykinesia or rigidity, difficulty in turning in bed upon awakening or getting out of bed, freezing gait, tremors, muscle cramps, or dysphagia [12]. Although several studies have documented the epidemiology, assessment, and management of SD and EMO in PwP [4, 10, 13–17], there is limited quantitative evidence on the impacts of SD and EMO on health-related quality of life (HRQoL) [7–9].

Newer treatments for advancing Parkinson’s disease (aPD) such as the novel continuous, subcutaneous infusions of levodopa- or apomorphine-formulations [18–21] and longer-established treatments such as levodopa/carbidopa intestinal gel [22–24] and deep brain stimulation [25–28] have been shown to improve SD and EMO in clinical trials [4]. To comprehensively assess and compare the value of aPD treatments, cost-effectiveness models must capture the full impact of aPD symptoms on HRQoL, as these evaluations are necessary to guide healthcare decision-makers in allocating resources and support treatment coverage. Many HTA bodies such as the National Institute for Health and Care Excellence (NICE), Haute Autorité de Santé, and Zorginstituut Nederland [29–31] prefer HRQoL to be measured using either the EQ-5D-5 L or EQ-5D-3 L descriptive systems [32, 33] to generate health state utility values (HSUVs) for economic modeling. Similarly, in the US, payers and HTA frameworks prioritize the use of EQ-5D for cost-effectiveness analyses [34]. Published literature confirms the validity and reliability of EQ-5D-5 L and EQ-5D-3 L in PD, showing that it adequately captures both motor and non-motor symptoms and their impact on daily functioning [35–38]. This supports its appropriateness for generating HSUVs in PD and its use in economic evaluations. However, a targeted review of the literature did not identify any HSUVs for SD or EMO in PD, and no suitable patient-reported outcome (PRO) or EQ-5D data are available. In such cases, vignette-based methods are commonly employed to estimate HSUVs [39–41].

Vignettes are often used to estimate HSUVs for medical conditions and treatment attributes when: (i) patients are difficult to access, (ii) there is a need to isolate the utility impact of specific attributes or symptoms, (iii) the health states of interest are acute and temporary, (iv) health states change or fluctuate over time, or (v) there is a need to estimate treatment process utilities [42]. In the context of PD, existing economic models have largely overlooked the impact specific to SD and EMO in PwP, resulting in an incomplete evaluation of therapies providing continuous, 24-hour symptom control. Therefore, the need was to isolate the impact of PD symptoms at night during sleep (i.e., SD) and in the early morning (i.e., EMO) separately from other aPD symptoms. In addition, EMO is transient and occurs briefly in the morning prior to the first dose of medication, and SD and EMO can fluctuate in unpredictable frequency and severity over time. Therefore, a vignette approach was considered appropriate.

This study aimed to estimate the HSUVs associated with SD and EMO in PwP via health state vignettes scored using the EQ-5D-5 L. It also aimed to further quantify the burden of SD and EMO in PwP.

## Methodology

### Study design

The study was a cross-sectional, online survey study with primary data collection in PwP. The study population consisted of adults aged 30 years or older, residing in the United States (US) or the United Kingdom (UK), who self-reported: having been diagnosed with PD for 5 years or more, experiencing 2 or more hours per day of OFF time─which indicates they met proxy criteria for aPD [43]─and being on oral PD medications. People were excluded if they received a diagnosis of atypical or secondary parkinsonism or have taken or are taking medicine for Alzheimer disease or cognitive impairment at the time of survey completion. This study was conducted as an add-on survey to a discrete choice experiment (DCE) study investigating treatment preferences of PwP. This add-on survey was fielded to individuals who agreed to be recontacted after engagement in and completion of the DCE, as well as newly recruited PwP, who did not participate in the DCE [44]. Participants were recruited by an online recruitment vendor (Global Perspectives) using its network of patient panels and were compensated according to current fair market value. Participants were recruited through primary care physicians and neurologists. Potential participants were contacted by telephone and unique survey links were provided to interested participants. The same link could not be used more than once. Centralized ethics approval was obtained from Salus IRB on 4 January 2024. All procedures followed guidelines outlined in the Declaration of Helsinki and good clinical practice of the International Conference on Harmonisation.

NICE recommends having a sample of patients or general population complete the EQ-5D, based on vignette descriptions of required health states when direct EQ-5D data are unavailable from relevant studies or literature. Patients were selected over general population as patients have more experience of aPD health states and patient-reporting of health on the EQ-5D represents the gold standard for health reporting across many HTA agencies [29, 34]. Prior studies using this approach have captured meaningful distinctions between health states with small sample sizes between 36 and 73 participants [45–47]. Therefore, this study aimed to recruit approximately 75 participants.

### Vignette and survey design

Four vignettes were developed to represent these health states:

- Vignette A: No SD or EMO (baseline vignette)
- Vignette B: SD (without EMO)
- Vignette C: EMO (without SD)
- Vignette D: Both SD and EMO

Insights from PwP, their care partners (CPs), and clinicians informed vignette development, as did validated questionnaires and other relevant literature, following best practice guidelines on the conduct of vignette studies [42]. One-on-one semi-structured concept elicitation interviews were conducted, as part of the connected DCE study, in two waves with a total of 19 PwP and CPs, using open-ended questions to understand experiences with aPD, including SD and EMO. Interviews were conducted with participants from three countries, and a sample with a range of demographic and clinical characteristics was sought to mitigate bias due to convenience sampling. Many interviewees mentioned experiences of SD and EMO, and these discussions were used to develop draft vignette descriptions focused on these elements of aPD [48]. Symptom descriptions were refined by reviewing the content and item wording of validated questionnaires assessing SD and EMO, such as the Parkinson’s Disease Sleep Scale version 2 (PDSS-2) [49] and the 7-item card for EMO screening in PD patients [12] (no PRO data was collected as part of the interviews). Vignettes were reviewed and refined by clinicians to ensure they were reflective of the states they intended to describe and presented in patient-friendly language.

**Fig. 1:**
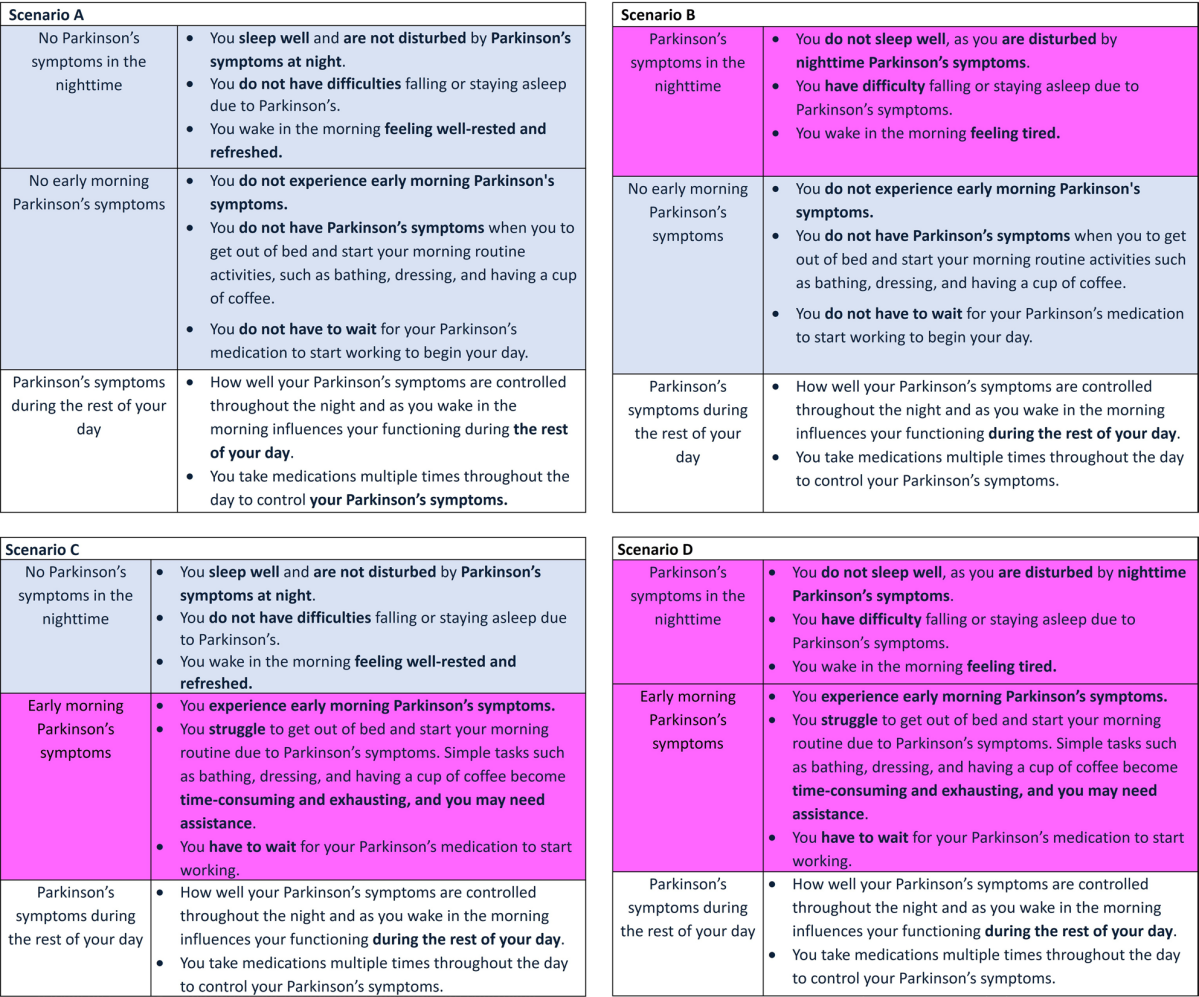
Vignettes

Vignettes were designed to cover a 24-hour period, split into 3 sections (Fig. 1). Each unlabeled vignette started with a section describing PD symptoms during the nighttime (SD), followed by a section describing PD symptoms in the morning (EMO), with symptoms in these sections varying across vignettes. A third section represented PD symptoms during the remainder of the day; this section was identical across the 4 vignettes to ensure participants imagined the remainder of the day uniformly and to avoid participants’ making assumptions that could influence results. Moreover, color coding and bold text were utilized to assist participants in identifying the differences between vignettes [50] (Fig. 1).

The survey began with a series of screening questions to determine eligibility. Eligible individuals were directed to provide informed consent, after which they were taken to the survey. The survey first asked participants to read and rank the vignettes from most to least preferred health state. This served to familiarize participants with the vignettes and to make them think about the relative severity of the vignette states. After completing the ranking exercise, participants were shown 1 vignette at a time. After reading each vignette, participants were asked to complete a modified version of the EQ-5D-5 L, by imagining how they would rate their health “on the entire day described above” by the vignette. Permission to modify the recall period of the EQ-5D-5 L was granted by the EuroQol Foundation.

The EQ-5D-5 L consists of 2 parts: a descriptive system with 5 dimensions (mobility, self-care, usual activities, pain/discomfort, and anxiety/depression), each rated on 5 levels from “no problems” to “extreme problems/unable to,” and a visual analog scale (VAS), which asks people to rate their health between 100 (best health you can imagine) and 0 (worst health you can imagine) [51]. To prevent ordering effects, vignettes were presented in a randomized order [52, 53].

The PDSS-2 instrument was then completed. The PDSS-2 is a validated questionnaire used to assess SD frequency in PwP, covering 15 items across 3 domains: “disturbed sleep,” “motor symptoms at night,” and “PD symptoms at night” [49]. Each item is rated from 0 (never) to 4 (very often), yielding domain scores ranging from 0 to 20 and a total score ranging from 0 to 60, with higher scores indicating greater sleep disturbance. PDSS-2 scores were also used to identify participants with a low (PDSS-2 < 18) versus high (PDSS-2 ≥ 18) burden of SD [54]. Further questions assessed the humanistic and clinical burden of SD and EMO in PwP, and clinical and demographic characteristics were captured.

The draft survey was pretested via one-on-one online interviews in a convenience sample of the general population. Pretests examined the feasibility of the survey and presentation of the vignettes in preparation for the launch of the online survey.

#### Data quality assurance

Respondents who answered the vignette section in an unreasonably short amount of time (less than 240 s to complete the first reading of the vignettes, the ranking task, and the 4 vignette EQ-5D-5 L completions) or who were suspected of inattention to the tasks due to illogical ordering of the extreme vignettes in the ranking exercise (Vignette D ranked as preferable to Vignette A) or in the VAS (Vignette A rated as worse than Vignette D) were excluded and replaced by new participants to ensure data quality.

### Statistical analysis

Descriptive statistics (mean, standard deviation, count, frequencies) were used to analyze sociodemographic and clinical characteristics, EQ-5D-5 L utility and VAS scores, as well as the humanistic and clinical burden of SD and EMO, burden of falls and CP-related factors. Data analysis was carried out using R version 4.2.2 and RStudio (Posit Software, Version 2023.03.1) [55, 56].

EQ-5D-5 L responses were obtained for the 4 vignette health states and converted into utility values using the US EQ-5D-5 L value set [57]. US value set was applied as these HSUVs were intended for use in an economic model from the US healthcare payer perspective. Disutility for SD, EMO, and EMO + SD was calculated by subtracting the EQ-5D-5 L utility for that health state from the baseline utility (Vignette A: No SD or EMO), as shown below.

$$\begin{aligned}\:{Disutility}_{SD,\:\:\:No\:EMO}=&{U}_{B\:(SD,\:\:\:No\:EMO)\:}\:\\&-\:{U}_{A\:(No\:SD,\:\:\:No\:EMO)}\:\:\:\:\:\:\:\:\:\:\:\end{aligned}$$

$$\begin{aligned}\:{Disutility}_{No\:SD,\:\:\:EMO}=\,&{U}_{C\:(No\:SD,\:\:\:EMO)}\:\\&-\:{U}_{A\:(No\:SD,\:\:\:No\:EMO)}\:\:\:\:\:\:\:\:\:\:\:\:\:\:\:\end{aligned}$$

$$\begin{aligned}\:{Disutility}_{SD,\:\:\:EMO}=\,&{U}_{D\:(SD,\:\:\:EMO)}\\&-\:\:{U}_{A\:(No\:SD,\:\:\:No\:EMO)}\:\:\:\:\:\:\:\:\:\:\:\:\:\:\:\:\:\:\:\:\:\:\:\:\:\:\:\end{aligned}$$

Given the sample size, a few outliers had a great impact on EQ-5D-5 L responses. Rescaling was used to limit the impact of outliers on results. The HSUVs were rescaled, using the formula below [58], by adjusting the reported utilities based on the minimum and maximum utilities of Vignette A and Vignette D observed in the data, respectively, to mitigate the impact of outlier responses. VAS scores were also rescaled following the same method.

$$\:{U}_{re-scaled}=\frac{{U}_{reported}-\:{U}_{\mathrm{m}\mathrm{i}\mathrm{n}\left(D\right(SD,\:\:\:EMO\left)\right)}}{{U}_{\mathrm{m}\mathrm{a}\mathrm{x}\left(\mathrm{A}\right(No\:SD,\:\:\:No\:EMO\left)\right)}-\:{U}_{\mathrm{m}\mathrm{i}\mathrm{n}\left(D\right(SD,\:\:\:EMO\left)\right)}}\:\:\:\:\:\:\:\:\:\:\:\:\:\:\:\:\:\:\:\:\:\:\:\:\:\:\:\:\:\:\:\:$$

## Results

### Respondent characteristics

The survey was completed by 99 PwP across the UK and US from February to June 2024. However, 24 participants were excluded following the data quality check. Participants were excluded because they completed the vignette section too fast (*n* = 9), due to illogical ordering of the extreme vignettes in the ranking (*n* = 7) and in the VAS exercise (*n* = 8). Therefore, the final sample consisted of 75 PwP.

Respondents had an average age of 64.32 years (standard deviation (Sd) = 9.61). The majority identified as male (52.00%), were White (62.67%), and resided in the US (78.66%). Most participants reported high levels of education, with 24.00% holding a college degree and 21.33% possessing an advanced or university degree. Almost all participants (90.66%) had someone providing caregiving assistance. This was commonly a spouse/partner (44.12%) or another family member (29.41%). CPs on average provided nearly 30 h of assistance/week, and commonly (72.05%) supported PwP in the early morning with medication management (Table 1).

**Table 1:** Sociodemographic characteristics

Variable	Respondents (*N* = 75)
**Country of residence, n (%)**	
US	59 (78.66%)
UK	16 (21.33%)
**Age (years)**	
Mean (Sd)	64.32 (9.61)
**Gender**,** n (%)**	
Male	39 (52.00%)
Female	35 (46.66%)
Prefer not to answer	1 (1.33%)
**Race**,** n (%) [US]**	
White	37 (62.71%)
Black or African American	8 (13.56%)
Native Hawaiian or Pacific Islander	2 (3.39%)
American Indian or Alaska native	1 (1.69%)
Asian	1 (1.69%)
Other	1 (1.69%)
Prefer not to answer	9 (15.25%)
**Hispanic**,** Latino or Spanish origin**,** n (%) [US]**	
Yes	7 (11.86%)
No	43 (72.88%)
Prefer not to answer	9 (15.25%)
**Ethnic group**,** n (%) [UK]**	
White	10 (62.50%)
Asian or Asian British	2 (12.50%)
Black or African or Caribbean or Black British	2 (12.50%)
White and Black Caribbean	1 (6.25%)
**Highest level of education achieved**, **n (%) [US]**	
High school graduate (includes GED)	19 (32.20%)
Some college or technical school	15 (25.42%)
College graduate	15 (25.42%)
Advanced or graduate/post-graduate degree	7 (11.86%)
Prefer not to answer	3 (5.08%)
**Highest level of education achieved**, **n (%) [UK]**	
Secondary school	3 (18.75%)
Vocational qualification	1 (6.25%)
College	3 (18.75%)
University degree (bachelor’s or master’s degree or PhD)	9 (56.25%)
Prefer not to answer	0 (0.00%)
**Living situation**,** n (%)**	
Live with a spouse/partner	42 (56.00%)
Live with other family member(s) and/or friend(s)	25 (33.33%)
Live at home alone	7 (9.33%)
Live in an assisted living or long-term care facility	1 (1.33%)
**Care partner relationship**,** n (%)**	
A spouse or partner	30 (44.12%)
Other family member	20 (29.41%)
Paid or professional caregiver	7 (10.29%)
Child or children	6 (8.82%)
Friend	5 (7.35%)
**Assistance from the care partner**,** hours per week**	
Mean (Sd)	29.55 (26.75)
**Activities in which the person with aPD needs support from care partner in the early morning**,** n (%)**	
Managing medication	49 (72.05%)
Transferring and walking	28 (41.17%)
Bathing, grooming and dressing	27 (39.70%)
Toileting and eating	20 (29.41%)

aPD = advancing Parkinson’s disease, GED = General Educational Development, IQR = interquartile range, Sd = standard deviation

1: 4 persons with aPD recruited ad hoc were not presented with this question

On average, respondents were diagnosed with PD 9.82 years ago (Sd = 4.63) and experienced 3.91 h (Sd = 2.69) of OFF time/day in the past week (Table 2). All respondents reported currently being on an oral medication, with most (81.66%) taking pills for PD at least 3–4 times/day, and being somewhat satisfied with their current PD treatment (42.25%). While most respondents reported being able to walk without assistance (47.89%), many (38.03%) reported their health as “fair” and no one reported their health as “excellent” (Table 2).

**Table 2:** Clinical characteristics

Variable	Respondents (*N* = 75)	
**Duration of diagnosis (Parkinson’s disease), years**		
Mean (Sd)		9.82 (4.63)
**Comorbid conditions other than PD**,** n (%)**		
Arthritis		18 (24.00%)
Depression, anxiety, or other psychiatric disorder		16 (21.33%)
Asthma, emphysema, or other pulmonary condition		11 (14.66%)
Cardiovascular disease		11 (14.66%)
Type 2 diabetes		9 (12.00%)
Gastrointestinal disease		7 (9.33%)
History of stroke		6 (8.00%)
Multiple sclerosis		2 (2.66%)
**OFF time/day in the past week**,** hours**^**2**^		
Mean (Sd)		3.91 (2.69)
**Ability to walk and get around**,** n (%)**^**1**^		
I am able to walk on my own without support from any walking aids.		34 (47.89%)
I usually use a walking aid, such as a cane or walker, to walk safely without falling. However, I do not usually need the support of another person.		22 (30.99%)
I usually use the support of another person to walk safely without falling.		8 (11.27%)
I have limited ability to walk and primarily use a wheelchair or assistance from another person to get around.		7 (9.86%)
**Treatments**,** n (%)**		**Current**
Oral medication (e.g., Sinemet, Rytary)		75 (100%)
Inhalator (i.e., Inbrija)		2 (2.66%)
Injection (i.e., Apokyn)		5 (6.66%)
Infusion (i.e., Duopa/Duodopa)		1 (1.33%)
Oral film (i.e., apomorphine)		2 (2.66%)
Transdermal patch/transdermal (i.e., Neupro)		1 (1.33%)
Deep brain stimulation		6 (8.00%)
**Frequency of taking PD oral pills**,** n (%)**^**1**^		
Once a day		4 (5.63%)
Twice a day		9 (12.67%)
3–4 times a day		46 (64.78%)
5–6 times a day		8 (11.26%)
7–8 times a day		1 (1.40%)
More than 8 times a day		3 (4.22%)
**Level of satisfaction with current PD treatment**,** n (%)**^**1**^		
Very dissatisfied		6 (8.45%)
Somewhat dissatisfied		11 (15.49%)
Neither satisfied nor dissatisfied		17 (23.94%)
Somewhat satisfied		30 (42.25%)
Very satisfied		7 (9.86%)
**Self-rated health**,** n (%)**^**1**^		
Excellent		0 (0.00%)
Very good		5 (7.04%)
Good		24 (33.80%)
Fair		27 (38.03%)
Poor		15 (21.13%)

aPD = advancing Parkinson’s disease, PD = Parkinson’s disease, Sd = standard deviation

1: 4 persons with aPD recruited ad hoc were not presented with this question; 2: OFF and ON times are based on individuals’ self-report

Cardiovascular diseases include angina, heart failure, history of heart attack, or other

### Burden of sleep disturbance

The mean total PDSS-2 score was 25.52 (Sd = 10.57), indicating a moderate level of overall SD among participants (Supplemental Table 1) [54]. Subscale scores provided further insights: “disturbed sleep” had a mean of 10.36 (Sd = 4.01), “motor symptoms at night” averaged 8.25 (Sd = 4.27), and “PD symptoms at night” averaged 6.91 (Sd = 3.70). Specific PDSS-2 items with the highest mean scores (more frequent disturbances) were “difficulties falling asleep” (2.09), “difficulties staying asleep” (2.17), “get up at night to pass urine” (2.12), and “tired and sleepy after waking in the morning” (2.24), indicating these are more common issues. Twenty-one (28%) individuals had PDSS-2 scores < 18 (low SD burden), while 54 (72%) had PDSS-2 scores ≥ 18 (high SD burden).

Overall, 81.32% of participants experienced interrupted sleep more than 1 night in the past week, with 38.67% reporting interrupted sleep at least 4 nights in the past week (Table 3). Only 4.00% did not report any interrupted sleep in the past week. Among participants who reported using melatonin to help with sleep (*n* = 47), over half (59.57%) took melatonin at least 4 nights in a week, with 25.53% using it nightly. Overall, 19 participants mentioned they had at least 1 fall during the nighttime in the past 12 months, with an average of 3.94 (Sd = 3.06) falls among those who reported a fall.

**Table 3:** Burden of sleep disturbance

Variable	Respondents (*N* = 75)
**Number of nights of interrupted sleep in the past week, n (%)**	
None: 0 nights	3 (4.00%)
Once a week: 1 night	11 (14.66%)
Every other night: 2–3 nights	32 (42.66%)
More frequently: 4–6 nights	16 (21.33%)
Every night: 7 nights	13 (17.33%)
**Frequency of melatonin usage in the past week for managing sleep problems**,** n (%)**	*n* = 47
Once a week: 1 night	2 (4.26%)
Every other night: 2–3 nights	17 (36.17%)
More frequently: 4–6 nights	16 (34.04%)
Every night: 7 nights	12 (25.53%)
**Frequency of falls during the night in the past 12 months**	
Number of persons reporting a fall at night, n (%)	19 (25.33%)
Number of times (in those who fell), Mean (Sd)	3.94 (3.06)
**Healthcare professional consultation for sleep disturbance in the past 12 months**, n (%)*	
General neurologist	31 (41.33%)
Family doctor/General practitioner	28 (37.33%)
Sleep disorder specialist or somnologist	18 (24.00%)
Parkinson’s nurse	18 (24.00%)
Movement disorder specialist	14 (18.66%)
Geriatrician	3 (4.00%)
None	12 (16.00%)
**Frequency of consultation for sleep disturbance in the past 12 months**,** Mean (Sd)**,** Min-Max**	
Family doctor/General practitioner	2.78 (1.39), 1–6
General neurologist	3.41 (2.04), 1–8
Movement disorder specialist	3.85 (2.76), 1–12
Sleep disorder specialist or somnologist	4.55 (2.99), 1–12
Geriatrician	6.33 (3.05), 3–9
Parkinson’s nurse	4.94 (3.28), 1–10

Max = maximum, Min = minimum, Sd = standard deviation

*Respondents could select more than one healthcare professional

While 84.00% of participants consulted a healthcare professional (HCP) about SD in the past year, only 41.33% consulted a general neurologist (GN), 18.66% consulted directly with a movement disorder specialist (MDS), and 24.00% spoke with a Parkinson’s nurse. SD issues were discussed in a higher proportion with family doctors/general practitioners (37.33%) and somnologists (24.00%). Geriatricians, Parkinson’s nurses, and somnologists had the highest number of visits, with averages of 6.33, 4.94, and 4.55 in the past 12 months, respectively. Out of the 75 participants, 37 (49.33%) had not consulted a GN or MDS regarding their SD.

### Burden of early morning OFF time

The majority of participants (88.00%) experienced EMO more than once a day in the past week (Table 4). Additionally, 41.33% of participants found their medication effective 1 to 2 h after taking it in the morning, while 12.00% had to wait more than 2 h for medication to take effect. Only 10.66% reported experiencing rapid symptom relief within half an hour.

**Table 4:** Burden of early morning OFF time

Variable	Respondents (*N* = 75)
**Frequency of EMO time in the past week, n (%)**	
None: 0 days	1 (1.33%)
Once a week: 1 day	8 (10.67%)
Every other day: 2–3 days	42 (56.00%)
More frequently: 4–6 days	12 (16.00%)
Every day: 7 days	12 (16.00%)
**Time it takes for the medication to become effective after it is taken when experiencing EMO**,** n (%)**	
Less than half an hour	8 (10.66%)
Between half an hour to 1 h	27 (36.00%)
More than 1 h but less than 2 h	31 (41.33%)
More than 2 h	9 (12.00%)
**Frequency of falls in the early morning in the past 12 months**	
Number of persons reporting a fall in the morning, n (%)	30 (40.00%)
Number of times (in those who fell), Mean (Sd),	3.23 (3.77)
**Healthcare professional consultation for EMO in the past 12 months**,** n (%)**	*n* = 74*
Family doctor/General practitioner	10 (13.33%)
General neurologist	39 (52.00%)
Movement disorder specialist	23 (30.66%)
Geriatrician*	3 (4.00%)
Parkinson’s nurse	14 (18.66%)
Other	2 (2.66%)
None	7 (9.33%)
**Frequency of consultation for EMO in the past 12 months**,	
**Mean (Sd)**,** Max - Min**	
Family doctor/General practitioner	2.8 (1.54), 6 − 1
General neurologist	3.12 (1.88), 8 − 1
Movement disorder specialist	4.04 (2.14), 8 − 1
Geriatrician	8.66 (0.57), 9 − 8
Parkinson’s nurse	4.14 (3.39), 10 − 1
Other	4.5 (2.12), 6 − 3

EMO = early morning OFF time, Max = maximum, Min = minimum, Sd = standard deviation

*1 out of the 75 participants reported to have not experienced EMO in the past week

Many participants experienced problems in the early morning including: trouble starting daily tasks (64.00%), muscle stiffness and difficulty moving out of bed (62.67%), slow movement or difficulty getting started (58.67%), trouble staying focused or thinking clearly (56.00%), pain that was worse in the morning (54.67%), or feeling unsteady or at risk of falling, especially when getting out of bed (54.67%) (Supplemental Table 2). Two-fifths of participants reported experiencing at least 1 fall in the early morning during the past 12 months, with an average of 3.23 falls (Sd = 3.77) among those who reported a fall.

GNs were the most consulted HCPs for EMO (52.00%), followed by MDSs (30.66%) and Parkinson’s nurses (18.66%). Geriatricians, despite being consulted by only 4.00% of respondents, had the highest mean number of consultations (8.66) in the past 12 months. Parkinson’s nurses (mean = 4.14) and MDSs (mean = 4.04) were also frequently consulted. 9% of PwP had not discussed their EMO with any HCP. Out of the 75 participants, 18 (24.00%) had not consulted a GN or MDS regarding their EMO.

### HSUVs for SD and EMO

**Fig. 2:**
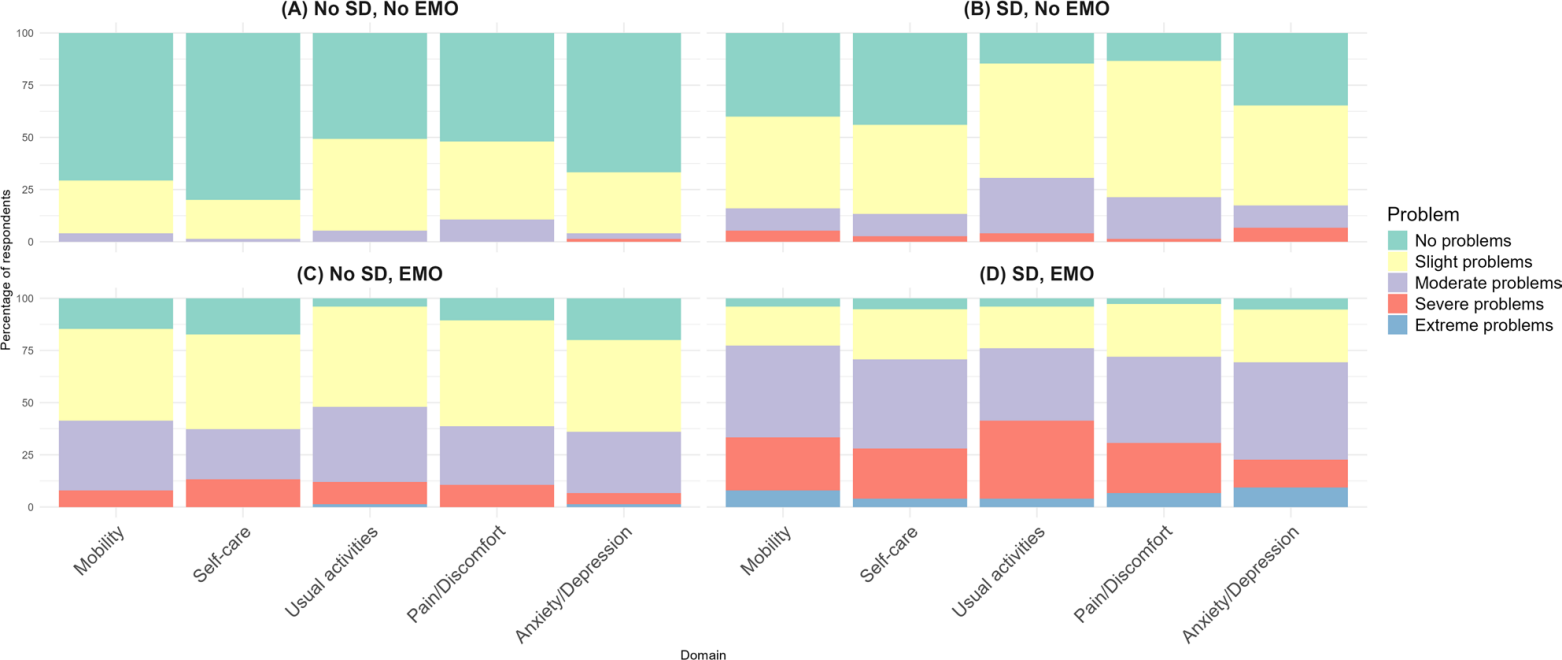
Distribution of EQ-5D-5 L item responses across vignettes. EMO = early morning OFF time, SD = sleep disturbance

Figure 2 presents the distribution of EQ-5D-5 L item responses across vignettes. Most respondents reported no problems in all 5 domains for Vignette A (No SD or EMO). However, slight problems were still common, particularly in usual activities (44.00%) and pain/discomfort (37.33%). There was a noticeable increase in reporting of moderate problems in Vignette B (SD, No EMO), especially in usual activities (26.67%) and pain/discomfort (20.00%). Severe issues also became evident, with 6.67% reporting severe problems with anxiety/depression. EMO symptoms described in Vignette C (No SD, EMO) resulted in more moderate and severe problems across all domains compared to Vignette B, with mobility showing the most significant deterioration. The combination of SD and EMO in Vignette D led to the highest reported severity of problems. Nearly half of respondents reported moderate to severe problems in multiple domains, and extreme problems were most prevalent in anxiety/depression (9.33%), mobility (8.00%), and pain/discomfort (6.67%). Only 4.00% of participants reported no problems with mobility and usual activities, respectively.

Table 5 presents the average rescaled and non-rescaled HSUVs and disutility values estimated for the different SD and EMO health states. When rescaling is applied, the health state without SD or EMO (Vignette A) provided the highest mean HSUV of 0.911 (Sd = 0.098). There are no corresponding disutility values for this vignette since it is the baseline state. When SD is present without EMO (Vignette B), the mean rescaled HSUV dropped to 0.796 (Sd = 0.128), resulting in a corresponding disutility of -0.115 (Sd = 0.130). EMO with no SD (Vignette C) resulted in a lower mean HSUV of 0.701 (Sd = 0.171), with a disutility of -0.210 (Sd = 0.170). This suggests that, on average, participants perceived EMO to have a greater impact on HRQoL than SD. The lowest HSUV was seen for the state with both SD and EMO (Vignette D), with a mean HSUV of 0.528 (Sd = 0.230) and a disutility of -0.384 (Sd = 0.230).

**Table 5:** Health state EQ-5D-5 L utilities and disutility values (rescaled and non-rescaled)

Vignette health state	Mean	Sd	SE	95% CI	Min	Max	Mean	Sd	SE	95% CI	Min	Max
	Utility values (Rescaled)						Utility values (Non-rescaled)					
A: No SD or EMO	0.911	0.098	0.011	0.889–0.934	0.587	1.000	0.861	0.154	0.018	0.825–0.896	0.35	1.000
B: SD (No EMO)	0.796	0.128	0.015	0.766–0.826	0.339	1.000	0.679	0.202	0.023	0.633–0.726	-0.04	1.000
C: EMO (No SD)	0.701	0.171	0.020	0.662–0.741	0.155	1.000	0.530	0.269	0.031	0.468–0.592	-0.329	1.000
D: SD + EMO	0.528	0.230	0.027	0.475–0.581	0.000	0.964	0.257	0.361	0.042	0.174–0.340	-0.573	0.943
	**Disutility values (Rescaled)**						**Disutility values (Non-rescaled)**					
B: SD (No EMO)	-0.115	0.130	0.015	-0.145- -0.086	-0.618	0.181	-0.182	0.204	0.024	-0.228- -0.135	-0.972	0.285
C: EMO (No SD)	-0.210	0.170	0.020	-0.249- -0.171	-0.845	0.042	-0.331	0.267	0.031	-0.392- -0.269	-1.329	0.066
D: SD + EMO	-0.384	0.230	0.027	-0.437- -0.331	-0.920	0.016	-0.604	0.361	0.042	-0.687- -0.521	-1.447	0.025

CI = confidence interval, EMO = early morning OFF time, Max = maximum, Min = minimum, Sd = standard deviation, SD = sleep disturbance, SE = standard error

US value set is used for US and UK participants

The non-rescaled values followed the same order across vignettes, but HSUVs were lower and disutility larger than the rescaled values due to the impact of outliers. The mean HSUV for the state without SD or EMO (Vignette A) was 0.861 (SD = 0.154), which is lower than the rescaled utility value. A mean HSUV of 0.679 (Sd = 202) was estimated when SD is present without EMO (Vignette B), resulting in a disutility of -0.182 (Sd = 0.204). A lower mean HSUV of 0.530 (Sd = 0.269) was observed for EMO with no SD (Vignette C), with a corresponding disutility value of -0.331 (Sd = 0.267). The mean HSUV dropped to 0.257 (Sd = 0.361) for the state with SD and EMO (Vignette D), with a mean disutility of -0.604 (Sd = 0.361). Supplemental Table 3 presents UK specific HSUVs and disutilities calculated using the Hernandez Alava et al. (2023) crosswalk algorithm as preferred by NICE [59].

The VAS responses for each health state and the differences across states were also calculated (Table 6). As the maximum VAS score in Vignette A was 100 and the minimum VAS score in Vignette D was 0, the non-rescaled and rescaled VAS values were identical. The order of magnitude of the mean VAS scores across vignettes was consistent with the order of the EQ-5D-5 L utility values. The mean VAS score for Vignette A was 81.01 (Sd = 9.15), followed by Vignettes B (65.85, Sd = 12.58), C (60.07, Sd = 13.62), and D (48.41, Sd = 16.51).

**Table 6:** Health state VAS values

VAS values							Difference in VAS scores between Vignette A and each vignette					
Vignette Health state	Mean	Sd	SE	95% CI	Min	Max	Mean	Sd	SE	95% CI	Min	Max
A: No SD or EMO	81.013	9.148	1.056	78.908–83.118	44	100	NA	NA	NA	NA	NA	NA
B: SD (No EMO)	65.853	12.575	1.452	62.960-68.746	33	96	-15.160	11.734	1.355	-17.859- -12.460	-53	12
C: EMO (No SD)	60.067	13.615	1.572	56.934–63.199	9	88	-20.947	13.316	1.538	-24.010- -17.882	-71	4
D: SD + EMO	48.413	16.513	1.907	44.614–52.212	0	80	-32.600	17.377	2.006	-36.597- -28.602	-96	-5

CI = confidence interval, EMO = early morning OFF time, Max = maximum, Min = minimum, NA = not applicable, Sd = standard deviation, SD = sleep disturbance, SE = standard error, VAS = visual analog scale

US value set is used for US and UK participants

Note: Rescaled and non-rescaled VAS values are the same

## Discussion

The findings of this study underscore the high prevalence and significant burden of SD and EMO on PwP, which are frequently undiagnosed [60], and offer a comprehensive understanding of the HRQoL impact and disutility of SD and EMO on PwP, assessed using a vignette approach.

SD was highly prevalent in PwP, with 96.00% of respondents experiencing interrupted sleep in the last week, and 72.00% of respondents experiencing a high SD burden (PDSS-2 score ≥ 18). Common issues included “difficulties falling asleep” and “staying asleep.” Despite the high prevalence of SD, 16.00% of participants had not consulted any HCP about it in the past year. PwP may not understand that their PD is the underlying cause of their SD, so this awareness needs to be raised to ensure they are discussing SD with their PD clinician team (e.g., MDS, GN, Parkinson’s nurses). The complexity of managing SD is heightened by its overlap with other PD symptoms (e.g., motor dysfunction, REM sleep behavior disorder) and comorbidities (e.g., anxiety, depression). The reliance on melatonin (used by 37.33% of participants at least 4 nights per week) highlights PwP seeking treatments to improve their sleep. However, it is imperative to treat the underlying cause of SD, which is primarily related to uncontrolled PD symptoms. It is therefore critical that MDSs and GNs assess PwP for poorly managed PD symptoms during sleep and optimize their current PD treatments or consider alternative treatments that provide uninterrupted, continuous 24-hour symptom management as appropriate.

EMO episodes were frequent and debilitating, with 98.67% of respondents experiencing EMO in the past week. The delayed onset of medication effectiveness exacerbates morning challenges, prolonging inactivity and discomfort. Falls were more common in the early morning (40.00%) than at night (25.33%) among respondents. Early morning activities, such as getting out of bed, often demand physical coordination, yet early morning is when PwP are most vulnerable to freezing of gait, imbalance, and stiffness, which together significantly increase the likelihood of falling. This highlights the need for treatment that not only provides PD symptom control overnight but also extends into the next day to allow PwP to wake up “ON,” reducing the risk of falls, improving morning safety, and allowing PwP to start their day unimpeded.

Estimated HSUVs and disutility values demonstrated the meaningful negative impact of SD and EMO on HRQoL. Participants perceived that EMO resulted in more severe problems than SD across EQ-5D-5 L domains, particularly in mobility, self-care, and usual activities, resulting in a larger disutility for EMO (-0.210) than SD (-0.115). This could be due to the fact that the EQ-5D dimensions of mobility, self-care, and usual activities are directly related to EMO (such as early morning mobility issues and delays/difficulties in being able to complete morning self-care and usual activities), whereas these EQ-5D dimensions are less directly related to SD.

To incorporate the disutility of SD and EMO into an economic model, each disutility should be weighted by the proportion of the population experiencing that specific symptom or symptom combination. Specifically, the disutility for SD is multiplied by the proportion of patients experiencing SD; the disutility for EMO is multiplied by the proportion of patients experiencing EMO; and the disutility for experiencing both SD and EMO is multiplied by the proportion of patients experiencing both. These weighted values can then be subtracted from the baseline HSUVs—gathered during clinical trials—to generate adjusted HSUVs that reflect the additional burden of SD and EMO.

Due to the high prevalence and significant detriment on HRQoL, it is important that PwP are assessed for PD symptoms impacting nighttime sleep and early morning periods. PwP and their CPs should be educated and empowered to discuss these challenges with their HCPs, especially MDSs, GNs, and PD nurses. For instance, implementing screening tools for SD and EMO during routine clinical visits could improve detection rates and facilitate earlier intervention. In addition, promoting the use of detailed sleep diaries, by teaching and encouraging PwP to regularly track their sleep patterns and related symptoms, can enhance symptom monitoring and support more effective communication between PwP and HCPs. For informed shared decision-making, it is important to allow for discussions of treatment optimization or consideration of alternative treatments that are proven efficacious in improving SD and EMO.

Emerging [18, 19] and established therapies [20, 22–28] have shown efficacy in extending ON time, reducing OFF episodes, improving PDSS-2 scores, and decreasing the frequency of morning akinesia. Advanced therapies, such as continuous infusions [18, 19] and deep brain stimulation [25, 26], have the potential to reduce EMO and SD. To ensure a more holistic assessment of their value, economic evaluations must incorporate the impact of SD and EMO on HSUVs, alongside related prevalence and healthcare resource utilization data. In NICE’s first technology appraisal of a Parkinson’s treatment, the 24 h continuous infusion of levodopa formulation (foslevodopa/foscarbidopa) was recognized for its potential benefit in improving sleep and nighttime symptom control [61, 62]. The committee highlighted that the benefits of better sleep in PD extend into the early waking hours. However, at the time of evaluation, there was no direct cost or utility data available to robustly model these benefits, underscoring the need for more comprehensive assessments in future economic evaluations [61].

Including the impact of treatments on SD and EMO in economic evaluations is essential to ensure that therapies providing continuous symptom control during the day and night are recognized for their full value in improving HRQoL. Early access to these treatments is crucial to prevent or mitigate the occurrence and severity of SD and EMO, ultimately enhancing long-term patient outcomes.

### Limitations

Vignette-based HRQoL studies present some limitations. First, utilities derived from vignettes may not perfectly align with the actual experiences of patients currently experiencing the state described. Second, the reliability of vignette-based utilities hinges on the accuracy and comprehensiveness of the health state descriptions, which are inherently limited, as they cannot include all possible aspects of the patient experience with PD. Third, vignettes cannot capture the full spectrum of patient experiences within a health state. As a result, the utilities derived from them may underestimate the heterogeneity associated with the condition. These limitations may be particularly poignant given the heterogeneity in PD symptoms, which can change hour by hour. Fourth, vignette-based methods for utility assessment are not standardized, and procedures for developing and valuing vignettes vary across studies [42].

Further potential methodological limitations include the required modification of the EQ-5D-5 L recall period, inclusion of time-based sections, and color coding in the vignettes and validation. The EQ-5D-5 L recall period of “today” was modified to “on the entire day described above” to enable vignette-based valuation. While this adjustment was approved by the EuroQol Foundation and required to align the instrument with the vignette-based approach, it reduces the comparability of resulting HSUVs with any obtained using the standard recall format. Time-based sections were included within the vignettes (rather than having separate vignettes per time period), which may have introduced contextual elements beyond HRQoL domains, influencing valuations. This approach was chosen as while SD and EMO occur at specific times of day, their impact often continues later into the day, which would be missed if vignettes were separated. Future studies could explore alternative designs to reduce potential bias. Color coding was utilized, following guidelines that color coding can be used when formatting vignettes, to maximize comprehension and reduce errors [42]; however, color coding introduces the potential for focusing effects. Lastly, although patient interviews were used to develop the vignettes, it was not possible to validate the final vignettes with patients. While external clinical experts validated the vignettes, ideally both groups would have been consulted at this stage to further strengthen content validity.

Other potential limitations include the feasibility of survey completion in aPD, failure to collect participants’ current EQ-5D-5 L scores, use of rescaling, potential for double counting and generatability of resulting HSUVs. Symptoms of aPD, such as tremor, rigidity, or cognitive difficulties, may have made it challenging for participants to complete the online survey without assistance, but other administration methods were not possible within this study. This study did not collect participants’ current EQ-5D scores, which could have provided a useful reference point to contextualize hypothetical responses; future research may incorporate this element to enhance interpretability. Rescaling of resulting HSUVs and disutilities was tested due to substantial heterogeneity in EQ-5D-5 L responses, with a few notable outliers in a small sample having a substantial impact on results. Both rescaled and non-rescaled results are presented, and users should consider which approach is most appropriate for their needs. Lastly, a potential limitation in the application of the obtained disutilities is that they might double-count the impact of SD and EMO if their impact is already reflected in baseline HSUVs. Potential users should consider how likely this is based on the source population and data collection schedule of their chosen baseline HSUVs. Finally, HSUVs and disutilities were calculated using the US value set and are therefore US-specific, with limited generalisability to other countries.

In the absence of direct EQ-5D responses from patients in each of these PD health states, this study attempted to align as closely as possible with the preferred methods for generating HSUVs. We recruited PwP and asked them to imagine their health in each of the vignettes rather than asking members of the general population, who would be expected to have substantially less experience of these types of symptoms and health states. Participants were asked to rate their perceived health in each vignette by responding to the EQ-5D-5 L, rather than by directly valuing the vignettes. This approach was taken to align utilities produced by this study with the gold standard of having patients directly report their own health on the EQ-5D-5 L [29, 34].

## Conclusion

SD and EMO are highly prevalent in PwP, which negatively affects their HRQoL. EMO has a greater impact than SD in isolation, as reflected particularly in the EQ-5D domains of mobility, self-care, and usual activities. The combined effect of SD and EMO exacerbates these challenges, further impairing HRQoL. For PwP experiencing SD and/or EMO, treatments providing continuous symptom control across the 24-hour day should be prioritized to maximize their HRQoL. Economic evaluations should account for the benefits of improving SD and EMO to ensure a more comprehensive assessment of treatment value. Therefore, this study provides specific disutility values for SD and EMO that can be incorporated into future health technology assessments, potentially influencing reimbursement decisions for treatments that address these symptoms.

## Supplementary Information

Below is the link to the electronic supplementary material.

Supplementary Material 1

## Data Availability

AbbVie studies that are available for data sharing are listed on Vivli. Data requestors should use the Vivli Data Request Form to request data package(s). Access to data is determined based on the business feasibility to support the request and the scientific merit of the research proposal.

## Abbreviations

aPD: Advancing Parkinson’s disease
CP: Care partner
EMO: Early morning OFF time
GN: General neurologist
HRQoL: Health-related quality of life
HSUV: Health state utility value
PD: Parkinson’s disease
PDSS-2: Parkinson’s Disease Sleep Scale version 2
PwP: People with Parkinson’s disease
Sd: Standard deviation
SD: Sleep disturbance
VAS: Visual analog scale
