# Supplementary material for: Burden and disutility of sleep disturbance and early morning OFF symptoms in people with advancing Parkinson’s disease: a vignette-based approach using the EQ-5D-5L
Source: J Patient Rep Outcomes. 2026 Apr 14;10:85. doi: 10.1186/s41687-026-01053-w (PMC13201744; doi:10.1186/s41687-026-01053-w)
Supplement: Supplementary file 1 — Supplementary Material 1 [file 41687_2026_1053_MOESM1_ESM.docx]

**Supplemental Material**

**Burden and disutility of sleep disturbance and early morning OFF symptoms in people with advancing Parkinson’s disease: a vignette-based approach using the EQ-5D-5L**

Supplemental Table 1 - PDSS-2 scores

|  | Mean score | Sd | Median | Ceiling (%)^a^  ^(no problems)^ | Floor (%)^a^  ^(very often problems)^ |
| --- | --- | --- | --- | --- | --- |
| 1. Sleeping well during the last week | 1.733 | 1.056 | 2 | 9.33% | 6.66% |
| 1. Difficulties falling sleep | 2.093 | 1.068 | 2 | 5.33% | 13.33% |
| 1. Difficulties staying asleep | 2.173 | 1.018 | 2 | 2.67% | 12.00% |
| 1. Restlessness of legs and arms at night | 1.773 | 1.085 | 2 | 12.00% | 8.00% |
| 1. Urge to move legs and arms | 1.720 | 1.073 | 2 | 16.00% | 5.33% |
| 1. Distressing dreams at night | 1.400 | 1.103 | 1 | 24.00% | 2.66% |
| 1. Distressing hallucinations at night | 0.733 | 0.963 | 0 | 58.67% | 0.00% |
| 1. Get up at night to pass urine (nocturia) | 2.120 | 1.039 | 2 | 4.00% | 10.66% |
| 1. Uncomfortable because of immobility | 1.787 | 1.044 | 2 | 8.00% | 6.66% |
| 1. Pain in arms and legs | 1.747 | 1.028 | 2 | 8.00% | 6.66% |
| 1. Muscle cramps in arms and legs | 1.533 | 0.920 | 2 | 12.00% | 2.66% |
| 1. Painful posturing of arms and legs in the morning | 1.693 | 1.052 | 2 | 12.00% | 5.33% |
| 1. Tremor on waking | 1.667 | 1.082 | 2 | 13.33% | 6.66% |
| 1. Tired and sleepy after waking in the morning | 2.240 | 0.984 | 2 | 2.67% | 12.00% |
| 1. Snoring or difficulties in breathing | 1.107 | 1.060 | 1 | 37.33% | 1.33% |
| Total Score: | 25.52 | 10.57 |  | 0.00% | 0.00% |
| Domain Scores: |  |  |  |  |  |
| Disturbed sleep  (1) Sleep during the last week,  (2) Difficulties falling sleep,  (3) Difficulties staying asleep,  (8) Get up at night to pass urine, and  (14) Tired and sleepy after waking in the morning | 10.36 | 4.01 | 10 | 0.00% | 0.00% |
| Motor symptoms at night  (4) Restlessness of legs and arms at night,  (5) Urge to move legs and arms,  (6) Distressing dreams at night,  (12) Painful posturing in the morning, and  (13) Tremor on waking | 8.253 | 4.272 | 8 | 0.00% | 1.33% |
| PD symptoms at night  (7) Distressing hallucinations at night,  (9) Uncomfortable because of immobility,  (10) Pain in arms and legs,  (11) Muscle cramps in arms and legs, and  (15) Snoring or difficulties in breathing | 6.907 | 3.702 | 6 | 1.33% | 0.00% |

Sd = standard deviation. The scores for each item assessed the frequency of symptoms, which ranged from 0 (never) to 4 (very often). The total score ranged from 0 (symptoms-free) to 60 (highly symptomatic). The PDSS-2 was further divided into 3 domain scores by summing individual item scores in groups of 5 (for a maximum score of 20): Domain scores consisted of “disturbed sleep,” “motor symptoms at night,” and “PD symptoms at night.”

^a^Ceiling refers to the % of people reporting no problems in each item and floor refers to the % of people reporting problems very often in each item,

Supplemental Table 2 - EMO impacts experienced in the early morning in the past week

| **Impacts experienced in the early morning in the past week, n (%)** | **Respondents (N=75)** |
| --- | --- |
| Having trouble starting daily tasks or regular activities | 48 (64.00%) |
| Muscle stiffness when waking up and difficulty moving out of bed | 47 (62.67%) |
| Moving more slowly or having a hard time getting started in the morning | 44 (58.67%) |
| Trouble staying focused or thinking clearly in the morning | 42 (56.00%) |
| Experiencing pain (more in affected side) that's worse in the morning | 41 (54.67%) |
| Feeling unsteady or at risk of falling, especially when getting out of bed | 41 (54.67%) |
| Feeling anxious or irritable in the early morning | 38 (50.67%) |
| Feeling down in the early part of the day | 38 (50.67%) |
| Significant shaking or tremors in the morning | 37 (49.33%) |
| Involuntary muscle movements like toe curling or abnormal posture | 35 (46.67%) |
| Needing or feeling the urge to go to the bathroom more often in the early morning | 29 (38.67%) |
| None of the above | 1 (1.33%) |

EMO = early morning OFF time

Supplemental Table 3 - Health state EQ-5D-5L utilities and disutility values using UK value set^1^ (rescaled and non-rescaled)

| **Vignette health state** | **Mean** | **Sd** | **SE** | **95% CI** | **Min** | **Max** | **Mean** | **Sd** | **SE** | **95% CI** | **Min** | **Max** |
| --- | --- | --- | --- | --- | --- | --- | --- | --- | --- | --- | --- | --- |
|  | **Utility values (Rescaled)** | | | | | | **Utility values (Non-rescaled)** | | | | | |
| A: No SD or EMO | 0.899 | 0.101 | 0.012 | 0.876 – 0.922 | 0.565 | 1.000 | 0.835 | 0.153 | 0.018 | 0.800 – 0.871 | 0.328 | 0.989 |
| B: SD (No EMO) | 0.788 | 0.115 | 0.013 | 0.761 – 0.814 | 0.372 | 1.000 | 0.666 | 0.174 | 0.020 | 0.626 – 0.706 | 0.035 | 0.989 |
| C: EMO (No SD) | 0.714 | 0.148 | 0.017 | 0.679 – 0.748 | 0.263 | 1.000 | 0.553 | 0.226 | 0.026 | 0.501 – 0.605 | -0.131 | 0.989 |
| D: SD+EMO | 0.553 | 0.215 | 0.024 | 0.503 – 0.602 | 0.000 | 0.9343 | 0.309 | 0.328 | 0.038 | 0.234 – 0.384 | -0.532 | 0.889 |
|  | **Disutility values (Rescaled)** | | | | | | **Disutility values (Non-rescaled)** | | | | | |
| B: SD (No EMO) | -0.112 | 0.125 | 0.014 | -0.083 – 0.140 | -0.589 | 0.185 | -0.169 | 0.189 | 0.022 | -0.213 - -0.126 | -0.895 | 0.281 |
| C: EMO (No SD) | -0.186 | 0.153 | 0.018 | -0.150 – 0.221 | -0.737 | 0.027 | -0.282 | 0.232 | 0.027 | -0.335 - -0.228 | -1.120 | 0.042 |
| D: SD+EMO | -0.346 | 0.214 | 0.025 | -0.297 – 0.396 | -0.883 | 0.000 | -0.526 | 0.325 | 0.037 | -0.601 – -0.452 | -1.341 | 0.000 |

CI = confidence interval, EMO = early morning OFF time, Max = maximum, Min = minimum, Sd = standard deviation, SD = sleep disturbance, SE = standard error

^1^Hernández Alava M, Pudney S, Wailoo A. Estimating the relationship between EQ-5D-5L and EQ-5D-3L: results from a UK population study. *Pharmacoeconomics*. 2023;41(2):199-207.
